# Phylogenetic analysis of the Australasian paralysis ticks and their relatives (Ixodidae: *Ixodes*: *Sternalixodes*)

**DOI:** 10.1186/s13071-017-2045-4

**Published:** 2017-03-02

**Authors:** Mackenzie L. Kwak, Ian Beveridge, Anson V. Koehler, Mallik Malipatil, Robin B. Gasser, Abdul Jabbar

**Affiliations:** 1 Department of Veterinary Biosciences, Melbourne Veterinary School, Faculty of Veterinary and Agricultural Sciences, The University of Melbourne, Werribee, Victoria 3030 Australia; 2 Centre for AgriBioscience, Department of Economic Development, Jobs, Transport and Resources, Bundoora, Victoria 3083 Australia; 3 La Trobe University, Bundoora, Victoria 3083 Australia

**Keywords:** *Ixodes*, *Sternalixodes*, Phylogeny, Molecular, Morphological, Tick

## Abstract

**Background:**

The Australasian paralysis ticks and their relatives, *Ixodes* Latrielle, subgenus *Sternalixodes* Schulze, are some of the most important ticks in the region. However, very little is known about their phylogenetic relationships. The aim of this study was to elucidate the evolutionary relationships of members of the subgenus *Sternalixodes* by undertaking phylogenetic analyses of morphological and molecular datasets.

**Methods:**

Adult females (*n* = 64) of *Sternalixodes*, including *Ixodes anatis* Chilton, 1904, *Ixodes confusus* Roberts, 1960, *Ixodes cornuatus* Roberts, 1960, *Ixodes cordifer* Neumann, 1908, *Ixodes dendrolagi* Wilson, 1967, *Ixodes hirsti* Hassall, 1931, *Ixodes holocyclus* Neumann, 1899, *Ixodes myrmecobii* Roberts, 1962 and *Ixodes trichosuri* Roberts, 1960, were examined morphologically. Subsequently, these *Ixodes* spp. were genetically characterised using cytochrome *c* oxidase subunit 1 (*cox*1) gene and the internal transcribed spacer 2 (ITS-2) of the rRNA. Both morphological and molecular datasets were analysed using various phylogenetic methods to assess the evolutionary relationship of various members of the subgenus *Sternalixodes*.

**Results:**

Phylogenetic analyses of the *cox*1 sequences and morphological characters datasets revealed that the Australian and Papuan *Sternalixodes* formed a distinct clade with the New Zealand member of the group *I. anatis* positioned basally, in a separate clade. *Ixodes holocyclus*, *I. cornuatus* and *I. myrmecobii* formed a distinctive clade in both the *cox*1 and morphological phylogenies. However, based on phylogenetic analysis of the ITS-2 data, *I. holocyclus* formed a separate clade whereas *I. cornuatus* and *I. myrmecobii* grouped in a different clade.

**Conclusions:**

The *cox*1 and morphological data suggest that the subgenus *Sternalixodes* is paraphyletic, and *I. anatis* is not a sternalixodid tick; hence, it should not be included in the subgenus. Based on the phylogenetic analyses of *cox*1 and ITS-2 sequences, it appears that *I. myrmecobii* and *I. cornuatus* are not subspecies of *I. holocyclus*. Although this study provided better insights into the taxonomic status of the subgenus *Sternalixodes*, a complete morphological and molecular (using multiple markers) phylogenetic analysis including all members of the subgenus would be required to more accurately elucidate the evolutionary relationships within the subgenus.

## Background

Ticks (Arachnida: Ixodidae) are important ectoparasites of humans and animals and can cause direct (e.g., paralysis, anaemia) as well as indirect (e.g., transmission of pathogens) effects on their hosts [1–3]. Ticks can be found on all continents and are known to feed on all types of terrestrial vertebrates, including mammals, birds, reptiles and amphibians [1, 3]. The life-cycle of ixodid ticks consists of four developmental stages, the egg, and three active parasitic stages, larva, nymph, and adult (male and female). Depending on the type (hard, Ixodidae or soft, Argasidae) and species of ticks, their life-cycle can vary significantly.

To date, 70 species (56 and 14 members of families Ixodidae and Argasidae, respectively) of ticks have been recorded from a variety of hosts (humans and domestic animals = 16; mammals, reptiles and birds = 54) from Australia [4]. Among different genera of hard ticks prevalent in Australia, *Ixodes* is arguably the most important and its members are known to transmit and/or harbour pathogens, including *Rickettsia australis* (the causative agent of Queensland tick typhus) in humans [5], and flaviviruses, bunyaviruses and *Cercopithifilaria johnstoni* Mackerras (Nematoda: Filarioidea) in wildlife [6–8]. Some *Ixodes* species such as *I. holocyclus* and *I. cornuatus* can also cause paralysis in humans, domestic animals, and wildlife [9]. *Ixodes holocyclus* is known to have an immunoeffectory action on humans, causing tick bite anaphylaxis [10].

Members of the genus *Ixodes* have not been intensively examined (e.g., phylogeny using combined morphological and molecular datasets, biology, life-cycle) within Australia. Few life-cycles have been elucidated and the bionomics of very few species are understood. Of the subgenera of *Ixodes*, *Sternalixodes* has received most attention. The subgenus comprises nine members, including *Ixodes anatis, I. confusus*, *I. cornuatus*, *I. cordifer*, *I. dendrolagi*, *I. hirsti*, *I. holocyclus*, *I. myrmecobii* and *I. trichosuri* [11]. However, the majority of studies have focussed on *I. holocyclus* and *I. cornuatus*, a species morphologically similar to *I. holocyclus*, aiming to determine their distribution [1, 12], morphological and molecular identification [1, 13] and phylogenetic relationships [14]. A number of questions therefore remain to be answered regarding the members of the subgenus *Sternalixodes*. For instance, the status of *I. myrmecobii* as subspecies of *I. holocyclus* as proposed by Roberts [1] needs to be tested. To date, Australian paralysis ticks and their relatives have not been analysed using morphological as well as molecular phylogenetics. Being an important subgenus, *Sternalixodes* requires systematic investigations to address a number of taxonomic questions regarding the validity of its members. Therefore, this study was designed to elucidate the evolutionary relationships of members of the subgenus *Sternalixodes* by undertaking phylogenetic analyses of morphological and molecular datasets.

## Methods

### Tick collection and morphological identification

**Fig. 1:**
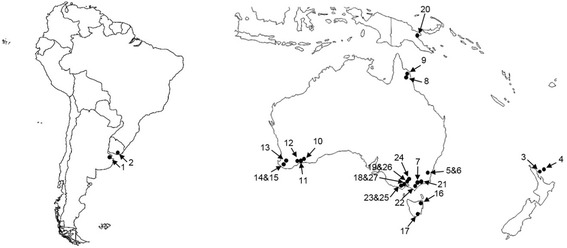
Collection sites for *Ixodes* species from Argentina, Australia, New Zealand and Uruguay, used in this study. Information linked to each unique *number* on the map is provided in Table 1

Female ticks (*n* = 74) used in this study were either available from The University of Melbourne (Ian Beveridge and Abdul Jabbar) or museums in Australia (South Australian Museum, Western Australian Museum, and Australian National Insect Collection), New Zealand (A. Heath, AgResearch, New Zealand), Papua New Guinea (Ifor L. Owen, National Veterinary Laboratory, Papua New Guinea) and South America (A. Guglielmone, Instituto Nacional de Technologia Agropecuaria, Argentina) (Fig. 1; Table 1). Following collection, each tick specimen was stored in 70% ethanol until used. Developmental stages of all species of *Sternalixodes* could not be examined as many are not yet described.

**Table 1:** Specimens of *Ixodes* spp. used in molecular studies, and their sources and collection sites

Species	Specimen voucher	Locality	Hosts/collection method	Source	Map code
*I. auritulus*	S63	Buenos Aires, Argentina	Collected via flagging	A. Guglielmone	1
	S64	Rocha, Uruguay	Collected via flagging	A. Guglielmone	2
*I. anatis*	S28	Auckland Zoo, New Zealand	*Apteryx mantelli*	A. Heath	3
	S29	Ponui Island, New Zealand	*Apteryx mantelli*	A. Heath	4
*I. holocyclus*	S1	Kioloa, NSW, Australia	Collected via flagging	This study	5
	S4	Kioloa, NSW, Australia	Collected via flagging	This study	6
	S37	Waygara, Vic, Australia	*Canis lupus familiaris*	This study	7
	S17	Wandecla NP, QLD, Australia	*Canis lupus familiaris*	This study	8
	S39	Atherton, QLD, Australia	*Canis lupus familiaris*	This study	9
*I. myrmecobii*	S26	Cape Le Grand NP, WA, Australia	Unknown	WAM	10
	S46	Munglinup, WA, Australia	*Homo sapiens*	WAM	11
	S56	Quaalup Station, WA, Australia	Unknown	WAM	12
	S44	Cranbrook, WA, Australia	*Homo sapiens*	WAM	13
	S25	Stirling Ranges NP, WA, Australia	Unknown	WAM	14
	S42	Stirling Ranges NP, WA, Australia	Unknown	WAM	15
*I. cornuatus*	S19	Mt William NP, Tas, Australia	*Vombatus ursinus*	ANIC	16
	S20	Acton Park, Tas, Australia	Unknown	This study	17
	S18	Bullengarook, Vic, Australia	*Canis lupus familiaris*	ANIC	18
	S41	Kinglake, Vic, Australia	*Canis lupus familiaris*	This study	19
*I. dendrolagi*	S14	Gondom, Papua New Guinea	*Dendrolagus matschiei*	This study	20
*I. trichosuri*	S23	Bellbird Creek, Vic, Australia	*Trichosurus caninus*	ANIC	21
	S21	Nowa Nowa, Vic, Australia	Unknown	ANIC	22
*I. hirsti*	S10	Anglesea, Vic, Australia	*Macropus giganteus*	This study	23
	S12	Mansfield, Vic, Australia	*Felis catus*	ANIC	24
	S9	Anglesea, Vic, Australia	*Macropus giganteus*	This study	25
*I. tasmani*	S68	Kinglake, Vic, Australia	Unknown	This study	26
	S69	Bullengarook, Vic, Australia	Unknown	This study	27

*Abbreviations*: *ANIC* Australian National Insect Collection, *NSW* New South Wales, *QLD* Queensland, *NP* National Park, *Tas* Tasmania, *WA* Western Australia, *WAM* Western Australia Museum, *Vic* Victoria

For morphological identification, each tick was examined using a dissecting microscope (Olympus, Japan). In addition, electron micrographs were taken using a Hitachi TM3030 Tabletop Scanning Electron Microscope, Germany. All Australian and Papuan tick species were identified using keys by Roberts [1]; whereas *I. anatis* specimens were identified following Hardwick [15] and *I. auritulus* Neumann, 1904 specimens were identified by A. Heath and A. Guglielmone.

One or two legs were removed from each specimen using flame sterilized forceps and stored in 70% ethanol for molecular work.

### DNA extraction, PCR amplification and DNA sequencing

Prior to DNA extraction, ethanol was removed and leg(s) of individual ticks were washed three times (30 min) in distilled H_2_O, and then ground using a plastic mortar. DNA was extracted using a DNeasy Blood and Tissue Kit (Qiagen, Hilden, Germany) following the protocol provided by the manufacturer.

Two loci (one mitochondrial and one nuclear ribosomal DNA) were PCR-amplified separately from each individual genomic DNA sample. The first locus (partial *cox*1 gene, ~850 bp) was amplified using the primers HCO2064 (5′-GGT GGG CTC ATA CAA TAA ATC C-3′) and HCOX1215 (5′-GCC ATT TTA CCG CGA TGA-3′); the second locus (partial second internal transcribed spacer, ITS-2; ~760 bp) was amplified employing primers ITS865 (5′-CTC GCC TGA TCG TGA GGT CG-3′) and ITS105 (5′-GGT CGA ATT GCC CCT CCT CG-3′) [14]. All PCRs were performed in a final volume of 50 μl, containing 10 mM Tris–HCl (pH 8.4), 50 mM KCl, 3.5 mM of MgCl_2_, 200 μM of deoxynucleotide triphosphate, 100 pmol of each primer and 1 U of Go*Taq* polymerase (Promega, Madison, WI, USA) under the following cycling conditions: 94 °C for 5 min (initial denaturation); 35 cycles of 94 °C for 30 s (extension), 48 °C (*cox*1) or 50 °C (ITS-2) for 30 s (annealing) and 72 °C for 50 s (extension), followed by final extension at 72 °C for 5 min. For each set of PCRs, negative (no-DNA) and positive (*I. holocyclus* DNA) controls were included. No amplification was detected in any of the negative control reactions at any time during the study. Amplicons (5 μl) were examined on 1.5% agarose gels stained with ethidium bromide. Gels were examined using transillumination and were photographed using a GelDoc system (BioRad, Hercules, CA, USA). If amplicons were not detected on agarose gel, then semi-nested PCRs were used as follows: HCOX1240 (5′-CCA CAA ATC ATA AAG ACA TTG G-3′) was used in conjunction with HCO2064 to amplify *cox*1 and ITS130 (5′-AGT TGT ACA TTG G-3′) in conjunction with ITS865 was used to amplify ITS-2. PCR cycling conditions for semi-nested PCRs were same as used above.

For each locus, amplicon(s) representing each *Ixodes* species were purified using shrimp alkaline phosphatase and exonuclease 1 [16] prior to automated DNA sequencing (ABI3730XL automatic sequencer at Macrogen Cooperation, South Korea). Sequencing of the *cox*1 and ITS-2 region was conducted using the primers HCO2064 and HCOX1215 or HCO2064 and HCOX1240 (*cox*1) and ITS865 and ITS105 or ITS865 and ITS130 (ITS-2), in separate reactions. The quality of each sequence obtained was appraised using the program Geneious Pro 6.5 (Biomatters Ltd., Auckland, New Zealand) [17]. Partial *cox*1 sequences were identified by local alignment comparison (set reading frames) using amino acid sequences conceptually translated using an online tool http://www.ebi.ac.uk/Tools/st/emboss_transeq/ from the respective loci of the reference sequence of *I. holocyclus* are available from GenBank.

### Phylogenetic analyses

For morphological phylogenetics, the character matrix was based on adult female specimens. All characters are morphological, collected by examining specimens using light and/or scanning electron microscopy. Characters that could not be scored with complete accuracy in some taxa were excluded from the analysis. Morphological data were analysed employing Maximum Parsimony (MP) in TnT [18], gaps were treated as missing characters, and bootstrap replicates and maximum trees were set at 10,000. In addition, data were analysed using Bayesian Inference (BI) by employing the Markov K model in MrBayes 3.2.6 [19–21]. Lset rates were set to gamma and coding was set to variable. Four simultaneous tree-building chains were used to calculate posterior probabilities (pp) for 2,000,000 generations, saving every 100th tree produced. Based on the final 75% of trees generated, a consensus tree was constructed. *Ixodes tasmani* Neumann, 1899 was used as the outgroup.

For molecular phylogenetics, nucleotide sequences were aligned using the MUSCLE V 3.8.31 program [22] and adjusted manually employing the program Mesquite V 3.03 [23]. Based on pairwise comparisons, sequence differences were calculated using the program MEGA 6.0. [24]. Two separate datasets representing *cox*1 and ITS-2 were compiled, together with reference sequences from GenBank [14, 25, 26]; *I. tasmani* and *I. uriae* White, 1852 were used as the outgroups, respectively. Both *cox*1 and ITS-2 sequences were aligned over 519 and 610 bp, respectively, and adjusted manually as described above. Phylogenetic analyses were performed on individual *cox*1 and ITS-2 datasets using Maximum Likelihood (ML), Neighbour-Joining (NJ) and BI methods. The ML and NJ analyses were performed using MEGA 6.0. and the nodes were tested for robustness with 10,000 bootstrap replicates. The data format was set to DNA and gaps were treated as missing data (10,000 bootstrap replicates, Max. trees was set at 10,000). The likelihood parameters for the BI (TIM2+I+G for pCXO1 and TVM+G for ITS-2) and ML (Tamura 3-parameter model for both *cox*1 and ITS-2) analyses were selected based on the Akaike Information Criterion (AIC) test in jModeltest v2.1.5 [27]. The BI was conducted, using Monte Carlo Markov Chain (MCMC) analysis in MrBayes 3.1.2. Four simultaneous tree-building chains were used to calculate posterior probabilities (pp) for 2,000,000 generations, saving every 100th tree produced. Based on the final 75% of trees generated, a consensus tree was constructed.

The phylogenetic trees produced for both morphological and molecular datasets were visually compared separately for concordance in their topologies.

## Results

### Morphological characterisation

Out of 74 individual specimens of female *Ixodes* examined, 64 belonged to the subgenus *Sternalixodes*, including *I. anatis* (*n* = 7), *I. dendrolagi* (*n* = 3), *I. cordifer* (*n* = 5), *I. cornuatus* (*n* = 5), *I. hirsti* (*n* = 10), *I. holocyclus* (*n* = 13), *I. myrmecobii* (*n* = 17) and *I. trichosuri* (*n* = 4); whereas, remaining 10 belonged to two subgenera *Endopalpiger* Schulze (*I. tasmani*; *n* = 6) and *Multidentatus* Neumann (*I. auritulus*; *n* = 4).

**Fig. 2:**
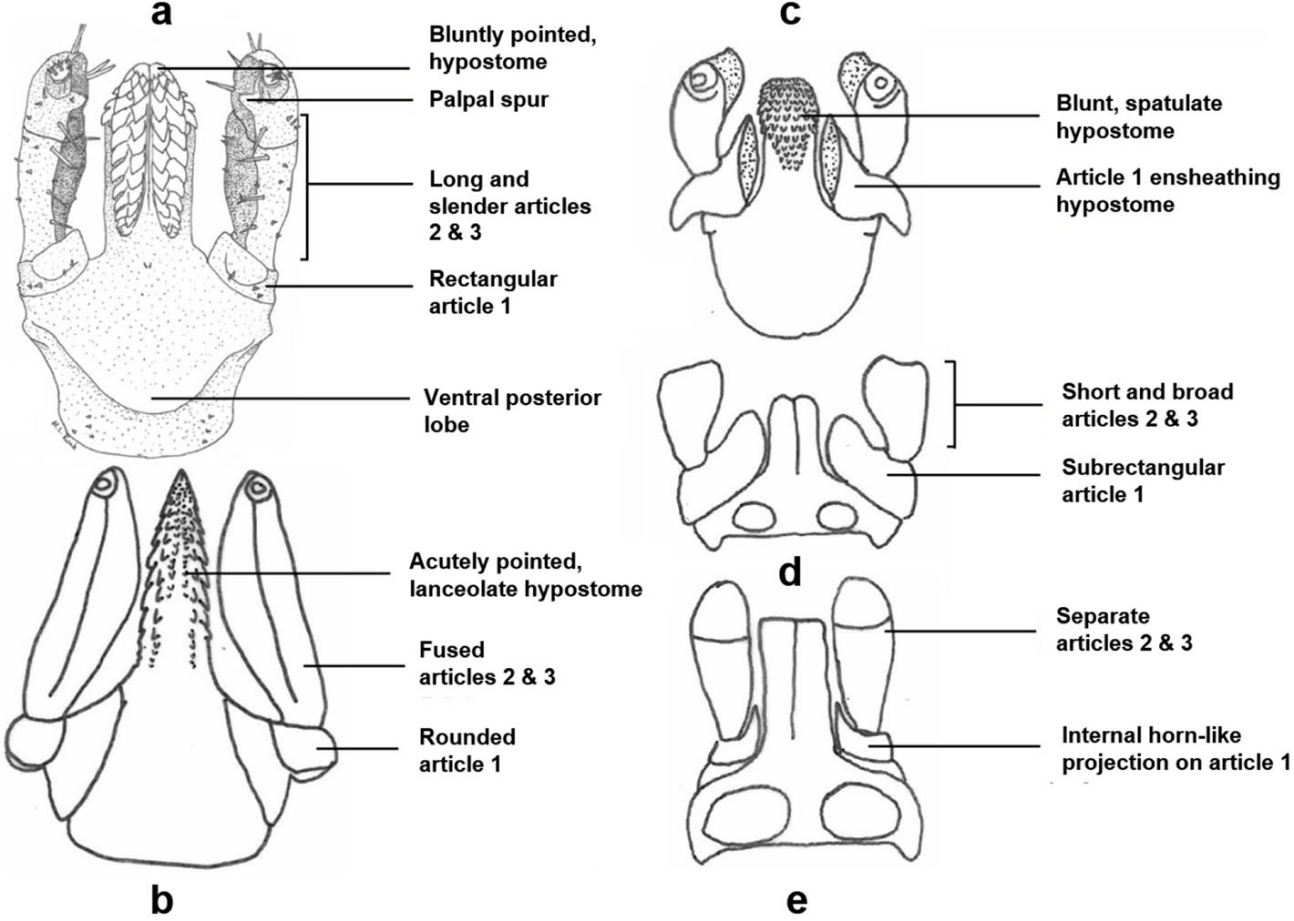
Principal features of the capitulum used as characters in Table 2. Capituli of **a**, *Ixodes cornuatus* (ventral view); **b**, *Ixodes hirsti* (ventral view)*;* **c**, *Ixodes tasmani* (ventral view); **d**, *Ixodes tasmani* (dorsal view), and **e**, *Ixodes auritulus* (dorsal view)

Character states are presented in Table 2, and the morphological data matrix is provided in Table 3. In addition, principal features of the capitulum used as characters are shown in Fig. 2.

**Table 2:** List of morphological characters (character numbers, name of character, character states)

No.	Feature or structure	Character states			
		0	1	2	3
1.	Hypostome - 1	lanceolate	spatulate		
2.	Hypostome - 2	blunt	bluntly-pointed	acutely pointed	
3.	Hypostome - 3	Not bilobed	bilobed		
4.	Dentition - 1	3/3 apically	4/4 apically	5/5 apically	
5.	Dentition - 2	only 3/3 mid-hypostome	4/4 and 3/3 mid-hypostome		
6.	Dentition - 3	2/2 basally	3/3 basally (1)		
7.	Palpal article 1-1	does not ensheath mouthparts	ensheathes basal portion of mouthparts		
8.	Palpal article 1-2	no internal horn-like projection	internal horn-like projection		
9.	Palpal article 1-3	rounded dorsally	rectangular dorsally	sub-rectangular dorsally	triangular dorsally
10.	Palpal articles 2 and 3-1	separate	faint suture present	between articles	fused
11.	Palpal articles 2 and 3-2	short and broad	long and slender		
12.	Palpal article 2 and 3-3	distal spur absent	distal spur present		
13.	Auriculae	absent	present		
14.	Cornua	absent	present		
15.	Ventral posterior lobe on basis capituli	absent	present		
16.	Porose areas	separated by equal to or less than half their width	separated by more than half their width		
17.	Median depression between porose areas	present	absent		
18.	Dorsal lateral carina(e) on basis capituli - 2	absent	not extending to base of hypostome	extending to base of hypostome	
19.	Dorsal carinae on basis capituli	median carina present	median carina absent		
20.	Ventral lateral carinae on basis capituli - 2	absent	not extending to base of hypostome	extending to base of hypostome	
21.	Ventral carinae on basis capituli - 3	no carinae	two carinae	three carinae	
22.	Scutum - 1	longer than wide	wider than long	as long as wide	
23.	Scutum - 2	lateral carinae absent	lateral carinae present		
24.	Scutum - 3	cervical grooves extending less than halfway down scutum	cervical grooves extending halfway or more down scutum		
25.	Scutum - 4	emarginations absent	emarginations present		
26.	Scapulae	absent	present		
27.	Sternal plate - (0), (1)	absent	present		
28.	Genital aperture	level with third intercoxal space	level with mid-fourth intercoxal space		
29.	Coxae I	external spur present	external spur absent		
30.	Coxae II	external spur present	external spur absent		
31.	Coxae III	external spur present	external spur absent		
32.	Coxae IV	external spur present	external spur absent		
33.	Syncoxae	Present	Absent		
34.	Ridges/rugosities - 1	absent on coxa I	present on coxa I		
35.	Ridges/rugosities - 2	absent on coxa II	present on coxa II		
36.	Ridges/rugosities - 3	absent on coxa III	present on coxa III		
37.	Ridges/rugosities - 4	absent on coxa IV	present on coxa IV		
38.	Anal groove	does not meet posteriorly	meets posteriorly

**Table 3:** Morphological character matrix of character states for each taxon used to construct morphological phylogeny

Species/State										1	1	1	1	1	1	1	1	1	1	2	2	2	2	2	2	2	2	2	2	3	3	3	3	3	3	3	3	3
	1	2	3	4	5	6	7	8	9	0	1	2	3	4	5	6	7	8	9	0	1	2	3	4	5	6	7	8	9	0	1	2	3	4	5	6	7	8
*I. auritulus*	0	0	0	2	1	1	0	1	3	1	1	0	1	1	0	0	0	0	0	0	0	0	0	1	0	0	0	0	1	1	1	1	0	0	0	0	0	0
*I. anatis*	0	0	1	0	0	0	0	0	1	0	1	1	1	0	1	0	0	0	0	0	0	1	0	1	0	0	0	0	1	0	0	0	0	0	0	0	0	0
*I. tasmani*	1	0	0	1	0	0	1	0	1	0	0	0	0	0	0	1	1	0	0	0	0	1	0	1	1	1	0	0	0	0	0	0	1	0	0	0	0	0
*I. holocyclus*	0	1	0	0	0	0	0	0	0	0	1	0	1	0	0	1	1	1	0	0	0	2	1	0	1	1	0	1	1	1	1	1	0	0	0	0	0	0
*I. cornuatus*	0	1	0	0	0	0	0	0	0	0	1	0	1	01	0	0	1	0	0	0	0	0	1	1	1	1	0	1	1	1	1	1	0	0	0	0	0	1
*I. myrmecobii*	0	1	0	0	0	0	0	0	0	0	1	0	1	1	0	1	1	1	0	1	1	0	1	0	1	1	0	0	1	1	1	1	0	0	0	0	0	1
*I. cordifer*	0	2	0	0	0	0	0	0	0	0	1	0	1	0	0	0	0	0	0	0	0	0	1	0	0	1	1	1	1	1	1	1	0	0	0	0	0	1
*I. dendrolagi*	0	2	0	0	0	0	0	0	0	0	1	0	1	0	0	0	0	1	1	1	1	0	1	1	0	1	1	1	1	1	1	1	0	1	1	1	0	1
*I. confusus*	0	2	0	0	0	0	0	0	0	0	1	0	1	0	0	0	0	2	1	2	2	0	1	1	1	1	1	1	1	1	1	1	0	1	1	1	1	1
*I. hirsti*	0	2	0	0	0	0	0	0	0	0	1	0	1	0	0	0	1	2	0	2	1	0	1	0	1	1	1	1	1	1	1	1	0	1	1	0	0	1
*I. trichosuri*	0	2	0	0	0	0	0	0	0	0	1	0	1	0	0	0	1	2	0	2	1	0	1	0	1	1	1	1	1	1	1	1	0	0	0	0	0	1

### Molecular characterisation

PCR amplification was successful for 27 (out of 64) genomic DNA samples extracted from individual tick specimens (Table 1). Considerable variation in the size (~650 to 750 bp) of amplicons (*n* = 15) for the ITS-2 was detected on agarose gel, whereas the amplicon size (~700 bp) for *cox*1 (*n* = 27) did not differ. DNA sequencing of amplicons for both loci revealed 27 and 15 unique sequences for *cox*1 and ITS-2, respectively. Sequence length, G+C content, pairwise differences and GenBank accession numbers for *cox*1 (KY213767–KY213793) and ITS-2 (KY213752–KY213766) sequences are given in Table 4. The length of *cox*1 sequences for each tick species was 674 bp, whereas that of ITS-2 ranged from 630 to 704 bp. Among various members of *Sternalixodes,* the highest genetic variation was detected in *I. cornuatus* (*n*umber of sequences = 4; pairwise differences 0.2–12%) followed by *I. anatis* (*n* = 2; 1.2%), *I. myrmecobii* (*n* = 6; 0.2–1.1%), *I. auritulus* (*n* = 2; 1.1%), *I. holocyclus* (*n* = 5; 0.2–0.9%), *I. hirsti* (*n* = 3; 0.2–0.3%) and *I. trichosuri* (*n* = 2; 0.3%) (Table 4). Based on ITS-2 sequences, multiple sequences for individual ticks were obtained only for *I. holocyclus* and *I. myrmecobii* and their pairwise differences were 1.4–8.6% and 0.8–3.6%, respectively (Table 4).

**Table 4:** Characteristics of sequences of *Ixodes* species determined in this study. GenBank accession numbers, G+C content and length of each sequence, and pairwise differences for each species with more than one specimens are provided

Species	Specimen voucher	*cox*1^*a*^				ITS-2^b^			
		GenBank accession no.	Length (bp)	G+C content (%)	Pairwise difference (%)	GenBank accession no.	Length (bp)	G+C content (%)	Pairwise difference (%)
*I. auritulus*	S63	KY213767	674	31.75	1.1	–	–	–	–
	S64	KY213768	674	31.75		–	–	–	–
*I. anatis*	S28	KY213769	674	31.90	1.2	KY213757	703	54.62	–
	S29	KY213770	674	31.90		–	–	–	–
*I. holocyclus*	S1	KY213782	674	32.20	0.2–0.9	KY213766	679	55.38	1.4–8.6
	S4	KY213783	674	32.05		KY213765	630	55.70	
	S37	KY213781	674	32.34		KY213756	684	55.40	
	S17	KY213779	674	32.49		KY213762	638	55.80	
	S39	KY213780	674	31.90		KY213755	676	55.47	
*I. myrmecobii*	S26	KY213784	674	30.86	0.2–1.1	KY213758	649	53.80	0.8–3.6
	S46	KY213785	674	31.16		KY213753	656	53.70	
	S56	KY213786	674	31.00		KY213752	657	53.60	
	S44	KY213787	674	31.00		–	–	–	
	S25	KY213788	674	30.70		KY213759	668	53.30	
	S42	KY213789	674	30.86		KY213754	647	53.80	
*I. cornuatus*	S19	KY213792	674	30.42	0.2–1.2	–	–	–	–
	S20	KY213793	674	30.12		–	–	–	–
	S18	KY213790	674	30.12		KY213761	654	53.36	–
	S41	KY213791	674	30.27		-	–	–	–
*I. dendrolagi*	S14	KY213776	674	30.70	–	KY213763	672	55.20	–
*I. trichosuri*	S23	KY213777	674	31.90	0.3	–	–	–	–
	S21	KY213778	674	31.90		KY213760	704	56.39	–
*I. hirsti*	S10	KY213773	674	33.10	0.2–0.3	–	–	–	–
	S12	KY213774	674	33.38		–	–	–	–
	S9	KY213775	674	33.23		KY213764	667	56.97	–
*I. tasmani*	S68	KY213771	674	32.20	10.9	–	–	–	–
	S69	KY213772	674	32.05		–	–	–	–

^a^ *cox*1: cytochrome *c* oxidase subunit 1

^b^ITS-2: second internal transcribed spacer

### Phylogenetic analyses

**Fig. 3:**
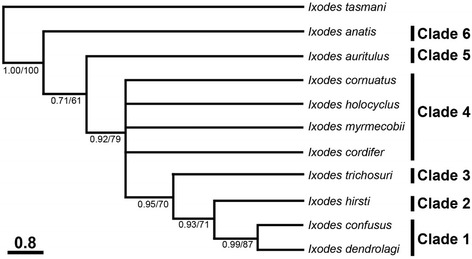
Morphological phylogram of specimens of *Sternalixodes* from Argentina, Australia, New Zealand and Uruguay. The relationships were inferred based on 38 morphological characters using Maximum Parsimony (MP) and Bayesian Inference (BI) methods. *Ixodes tasmani* was used as the outgroup. There was a concordance in the topology between this MP tree and that produced using BI (not shown). Nodal support (from *left* to *right*) is given as a posterior probability for BI and bootstrap values for MP. For simplicity, each clade number includes taxa in the preceding clade. The *scale*-*bar* indicates the number of inferred substitutions per character

The topology of the phylogenetic trees generated for morphological data employing BI and MP methods were similar (data not shown); hence, the MP tree is presented here, with nodal support values given for both methods (Fig. 3). The morphological phylogram showed six main clades, clade numbers including taxa of the preceding clade. *Ixodes confusus* and *I. dendrolagi* grouped together in clade 1, with moderate statistical support (posterior probability for BI: 0.99; bootstrap value for MP: 87%) (Fig. 3). *Ixodes hirsti*, *I. trichosuri*, *I. auritulus* and *I. anatis* each formed a clade (2, 3, 5 and 6, respectively), with no to high statistical support (Fig. 3). The common Australian paralysis tick, *I. holocyclus*, and *I. cordifer*, *I. cornuatus* and *I. myrmecobii* formed clade 4, with low to moderate statistical support (0.92, 79%).

**Fig. 4:**
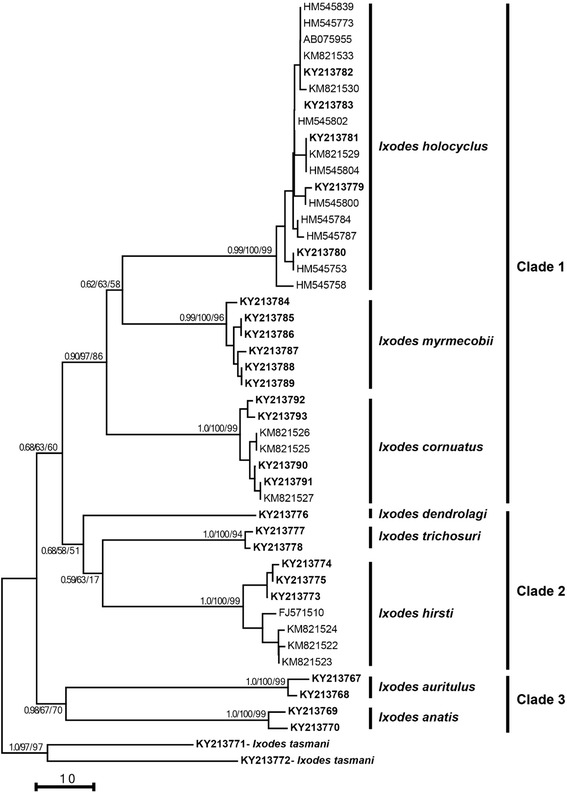
Genetic relationships of specimens of *Sternalixodes* from Argentina, Australia, New Zealand and Uruguay. The relationships were inferred based on phylogenetic analyses of the cytochrome *c* oxidase subunit 1 (*cox*1) sequence data determined herein (*bold*) using Bayesian Inference (BI), distance-based Neighbor Joining (NJ) and Maximum Likelihood (ML) methods. Previously published sequences of *Ixodes* species were obtained from GenBank (see accession numbers). *Ixodes tasmani* was used as the outgroup. There was a concordance in the topology between this NJ tree and those produced using BI and ML (not shown). Nodal support (from *left* to *right*) is given as a posterior probability for BI and bootstrap values for NJ and ML. The *scale*-*bar* indicates the number of inferred substitutions per nucleotide site

**Fig. 5:**
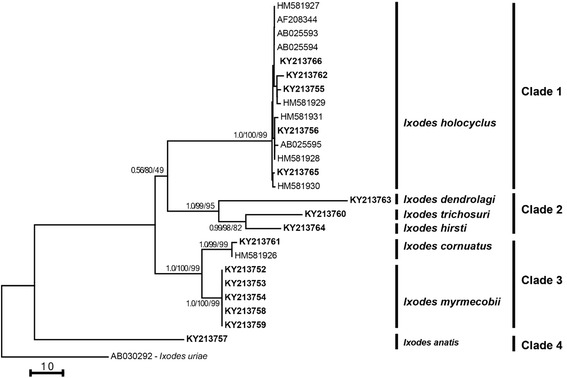
Genetic relationships of specimens of *Sternalixodes* from Argentina, Australia, New Zealand and Uruguay. The relationships were inferred based on phylogenetic analyses of the second internal transcribed spacer (ITS-2) sequence data determined herein (*bold*) using Bayesian Inference (BI), distance-based Neighbor Joining (NJ) and Maximum Likelihood (ML) methods. Previously published sequences of *Ixodes* species were obtained from GenBank (see accession numbers). *Ixodes uriae* was used as the outgroup. There was a concordance in the topology between this NJ tree and those produced using BI and ML (not shown). Nodal support (from *left* to *right*) is given as a posterior probability for BI and bootstrap values for NJ and ML. The *scale*-*bar* indicates the number of inferred substitutions per nucleotide site

Molecular phylogenetic analyses revealed that the topology of trees generated from the *cox*1 (aligned over 519 positions) and ITS-2 (608 positions) sequence data were similar using BI, NJ and ML (data not shown); hence, only the NJ trees for both loci are presented here (Figs. 4 and 5). The *cox*1 tree had three major clades (Fig. 4) in which *I. cornuatus*, *I. holocyclus* and *I. myrmecobii* formed Clade 1, with mixed statistical support (posterior probability for BI: 0.90; bootstrap value for NJ and ML: 97 and 86%). Individually, five *cox*1 sequences of *I. holocyclus* determined herein (GenBank accession nos. KY213779–KY213782) grouped together with those previously published from Australia, with strong statistical support (0.99, 100, 99%) (Fig. 4). All six *cox*1 sequences of *I. myrmecobii* grouped together with strong statistical support (0.99, 100, 96%), whereas four *cox*1 sequences of *I. cornuatus* found in this study formed two sub-clades with strong statistical support (1.0, 100, 99%) in which two sequences from Tasmania (KY213792 and KY213793) grouped outside the other two sequences from this study (KY213790 and KY213791) as well as previously published sequences (Fig. 4). Clade 2 contained *I. dendrolagi*, *I. hirsti* and *I. trichosuri* but without statistical support (0.68, 58, 51%; Fig. 4). However, individual sequences of *I. hirsti* determined here (KY213773–KY213775) formed a separate sub-clade compared with previously published sequences of this species. *Ixodes auritulus* and *I. anatis* formed Clade 3 with weak to moderate statistical support (0.98, 67, 70%) (Fig. 4).

The ITS-2 tree contained four major clades (Fig. 5). However, the composition of some clades was different from that found in the *cox*1 tree. For example, in the ITS-2 tree, Clade 1 contained only *I. holocyclus* with strong statistical support (1.0, 100, 99%) whereas *I. cornuatus* and *I. myrmecobii* formed a separate clade (Clade 3) with strong statistical support (1.0, 100, 99%) (Fig. 5). Similarly in the *cox*1 tree, *I. dendrolagi*, *I. hirsti* and *I. trichosuri* formed a separate clade (Clade 2) with strong statistical support (1.0, 99, 95%) whereas *I. anatis* formed a separate clade outside Australian and Papuan *Ixodes* spp. (Fig. 5).

## Discussion

This study addressed the evolutionary relationships amongst the species of *Sternalixodes*, using both morphological and molecular phylogenetic methods. The *cox*1 and morphological data suggest that the subgenus is paraphyletic with *I. anatis* which is congruent with a previous suggestion by Heath [28].

The topology was similar in the *cox*1 and morphological trees, with the Australian and Papuan *Sternalixodes* forming a distinct clade and the New Zealand member of the group *I. anatis* positioned basally, in a separate clade. *Ixodes holocyclus*, *I. cornuatus* and *I. myrmecobii* formed a distinctive clade in both the *cox*1 and morphological phylogenies. This pattern supports comments made by Roberts [1], who suggested that these three species were closely related and that *I. myrmecobii* and *I. cornuatus* may be subspecies of *I. holocyclus. Ixodes hirsti*, *I. trichosuri* and *I. dendrolagi* formed a separate clade distinct from the *I. holocyclus* species group. It is possible that the resulting tree would show *I. dendrolagi* forming a distinct clade with these northern species rather than grouping with *I. hirsti*. The *cox*1 phylogeny suggests that the outgroup *I. tasmani* contains a cryptic species based on the long branch lengths of the two *I. tasmani* samples (see Fig. 4) as previously proposed by Roberts [1].

While the morphological and *cox*1 trees showed similar topologies, there were some differences. The position of *I. auritulus* was different between the *cox*1 tree and the morphological tree, while *I. auritulus* and *I. anatis* formed a distinct clade in the *cox*1 phylogram. However, in the morphological tree *I. auritulus* formed a clade with the Australian/Papuan *Sternalixodes*. It is likely that the groups are only distantly related as *I. anatis*, *I. auritulus* and the Australian *Sternalixodes* are all morphologically and molecularly distinct. More extensive morphological and molecular phylogenetic analyses are required to adequately illuminate the evolutionary relationships between the three groups. This would require more extensive morphological character sets as well as the use of other molecular markers such as 16S, 28S or complete mitochondrial genomes.

Topological differences were also present between the two molecularly derived trees (see Figs. 4 and 5). The members of the *I. holocyclus* species group did not form a distinct clade in the ITS-2 tree as was seen in the *cox*1 tree, but instead formed two separate clades comprising *I. cornuatus* and *I. myrmecobii* in one and *I. holocyclus* in the other. The morphologically similar species, *I. holocyclus* and *I. cornuatus* grouped in highly divergent clades contrary to morphological evidence, as well as inferences from previous study of the interspecific relationship of these species by Song et al. [14]. This was likely due to the conserved nature of this marker in ticks. Song et al. [14] commented on the conserved nature of ITS-2 in *Sternalixodes* noting that intraspecific variation between *I. holocyclus* and *I. cornuatus* was as low as 0.19%. Despite suggestions that ITS-2 is suitable for inferring evolutionary relationships in ticks [14], it appears that it may not be suited for revealing the relationships between more distantly related species within subgenera.

Within the *cox*1 phylogeny, *I. hirsti* was divided into two distinct subclades (see Fig. 4). The GenBank sequences utilised were from ticks collected in South Australia [29], while the sequences obtained during this study were from Victorian specimens. This pattern in the *cox*1 sequences coupled with the geographic difference between the two groups suggests the group may be undergoing genetic differentiation.

The results of this study inform a number of historical questions and uncertainties concerning the subgenus *Sternalixodes*. Previously, Song et al. [14] used molecular techniques to assess the validity of the *I. holocyclus* species group. In the present study, based on the *cox*1 and ITS-2 trees, it appears that *I. myrmecobii* and *I. cornuatus* are not subspecies of *I. holocyclus*, each being a valid species. This contradicts the suggestion made by Roberts [1] that these two species may be subspecies of the widespread *I. holocyclus*. The results of this study are congruent with the results of Song et al. [14] and Jackson et al. [13]. However neither of these studies included the Western Australian species, *I. myrmecobii*.

The findings of this study also provided insights into the validity of *Sternalixodes* as a subgenus. The apparent paraphyletic status of the subgenus based on the position of *I. anatis* in the *cox*1 and morphological phylograms validates the suggestion by Heath [28] that the species should not be included in *Sternalixodes*. Heath [28] made the suggestion, citing the morphology of *I. anatis* as being incongruent with the morphological definition of *Sternalixodes*. Based on both morphological and *cox*1 data, it appears that *I. anatis* is not a sternalixodid tick and should not be included in the subgenus. This species does not meet the criteria of any of the subgenera of *Ixodes* defined by Clifford et al. [11]. However, Clifford et al. [11] noted that the classification of the subgenera of *Ixodes* was inaccurate in some situations, especially with regard to the subgenus *Ixodes*. An extensive examination of all existing subgenera using molecular and morphological data should be made in future to provide a more accurate hypothesis of the evolutionary relationships between the subgenera and the validity of the species within them. Based on the distinctive morphology of *I. anatis* and the fact that it does not meet the diagnostic requirements of any of the known subgenera of *Ixodes*, it may require the erection of a new subgenus. However more extensive molecular data should be accumulated and examined before this can occur.

Although seven of the nine species of *Sternalixodes* were examined in this study, molecular sequences and morphologically complete specimens were not located for *I. confusus* and *I. cordifer*. Although these species are most likely members of *Sternalixodes*, a complete molecular phylogenetic analysis including these species would be desirable to more accurately illuminate the evolutionary relationships within the subgenus.

As *I. myrmecobii* clusters within the *I. holocyclus* species group, a set of ticks known to cause paralysis, the question of its ability to also induce paralysis is raised. Tick induced paralysis has been extensively studied on the east coast of Australia; however, little information exists concerning ticks in Western Australia, let alone tick paralysis in Western Australia [9]. Studies of *I. myrmecobii* should be undertaken to determine if this species can induce paralysis. Roberts [30] and Kemp [31] noted that *I. hirsti* has been recorded to cause paralysis. Kemp [31] also proposed that all sternalixodid ticks may be capable of causing paralysis. As *I. hirsti* clustered with *I. trichosuri* and *I. dendrolagi* within the morphological and *cox*1 phylogeny, it is possible that these species may also be capable of inducing paralysis, however, this should be investigated.

## Conclusion

In conclusion, the *cox*1 and morphological data suggest that the subgenus *Sternalixodes* is paraphyletic, and *I. anatis* should not be included in this subgenus. Based on the phylogenetic analyses of *cox*1 and ITS-2 sequences, it appears that *I. myrmecobii* and *I. cornuatus* are not subspecies of *I. holocyclus*, each being a valid species. Although this study has improved insights into the taxonomic status of the subgenus *Sternalixodes*, a complete morphological and molecular (using multiple markers) phylogenetic analysis including all nine species of the subgenus would be desirable to more accurately illuminate the evolutionary relationships within the subgenus.

## Abbreviations

AIC: Akaike information criterion
BI: Bayesian inference
*cox*1: Cytochrome *c* oxidase subunit 1 gene
ITS-2: Internal transcribed spacer 2
MCMC: Monte Carlo Markov Chain
ML: Maximum likelihood
NJ: Neighbour-joining
